# Cognitive function in long term psychosis patients in a tertiary care hospital

**DOI:** 10.1192/j.eurpsy.2025.2264

**Published:** 2025-08-26

**Authors:** S. Shah, K. Patel, A. Bhandari, A. Porwal, N. Lalwani, K. Khetani, M. Parikh

**Affiliations:** 1 B.J. Medical College; 2 Department of Psychiatry, Civil Hospital, Ahmedabad, India

## Abstract

**Introduction:**

Psychosis refers to symptoms that are positive, disorganised,or negative and affects 3% of the population yet little information on long-term cognition is available.

**Objectives:**

To determine association between symptom severity,duration of disease and cognitive impairment.

**Methods:**

50 adult patients suffering from psychosis(DSM-5) for minimum 1 year and currently on at least 1 antipsychotic drug whose cognition and severity of symptoms were assessed by Montreal Cognitive Assessment test(MoCA) and Positive and Negative Syndrome Scale (PANSS) respectively were included in this cross sectional,cohort study.

**Results:**

Mean age of population was 41.5±12.33 years.Disorganised behaviour and speech were the prevalent core symptoms and risperidone was the choice of drug.

**Table:** 

Table 1	MALE	FEMALE	P value T-test	Overall mean
Total PANSS Score	68.2 ± 27.5	75.2 ± 34.9	0.430	71.1 ± 30.7
Positive PANSS	15.3 ± 7.67	17.4 ± 7.59	0.357	16.2 ± 7.63
Negative PANSS	17.2 ± 7.06	18.3 ± 8.92	0.646	17.7 ± 7.83
Generalised PANSS	35.6 ± 14.7	39.5 ± 20.6	0.434	37.2 ± 17.3

**Table:** 

Table 2	MALE (58%,n=29)	FEMALE(42%,n=21)	OVERALL(n=50)
Mild cognitive impairment(CI)	34%(17)	14%(7)	48%(24)
Moderate CI	24%(12)	20%(10)	44%(22)
Severe CI	0%	8%(4)	8%(4)

Symptom severity was mild and more negative symptoms were prominent (composite score: -1.48±6.47) (Table 1 PANSS Score) and an overall moderate cognitive impairment(16.5±4.46, table 2) was seen in population. Females showed a significantly lower MoCA score as compared to males (14.9±4.8 vs 17.65 ± 3.78, p=0.03) implying more cognitive decline (image 3).There is a strong, negative, linear correlation between MoCA and PANSS scores (r= -0.688, p<0.001) wherein all domains(image 1)except memory were negatively correlated to PANSS. Duration of illness showed a moderate, positive,linear and weak, negative, linear correlation with PANSS( r= 0.417, p =0.003) and MoCA(r = -0.314, p= 0.026) respectively. PANSS items were negatively correlated to MoCA in positive, negative and generalised items except delusions, blunted effect and tension(image 2).

**Image 1:**

**Figure:**
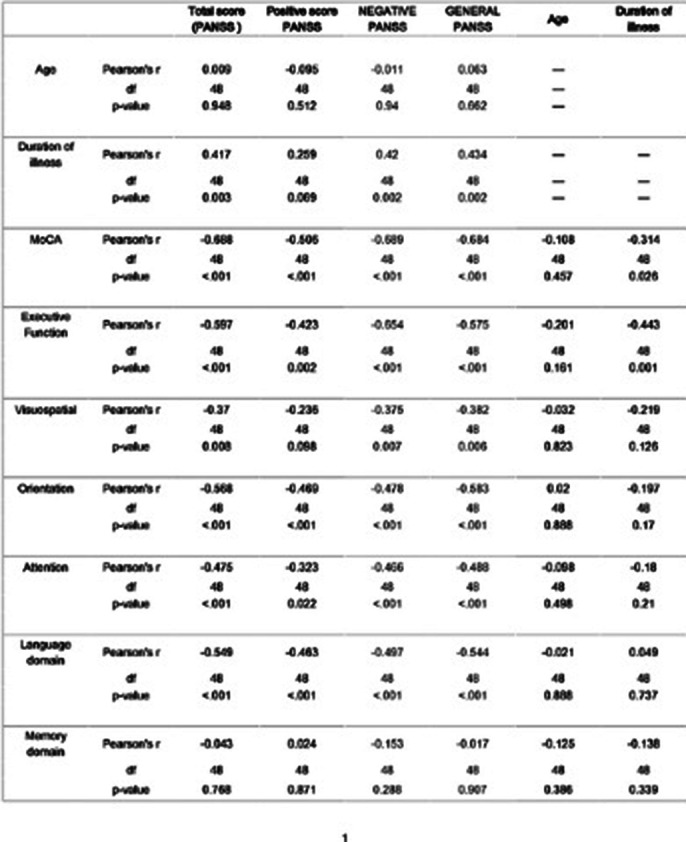

**Image 2:**

**Figure:**
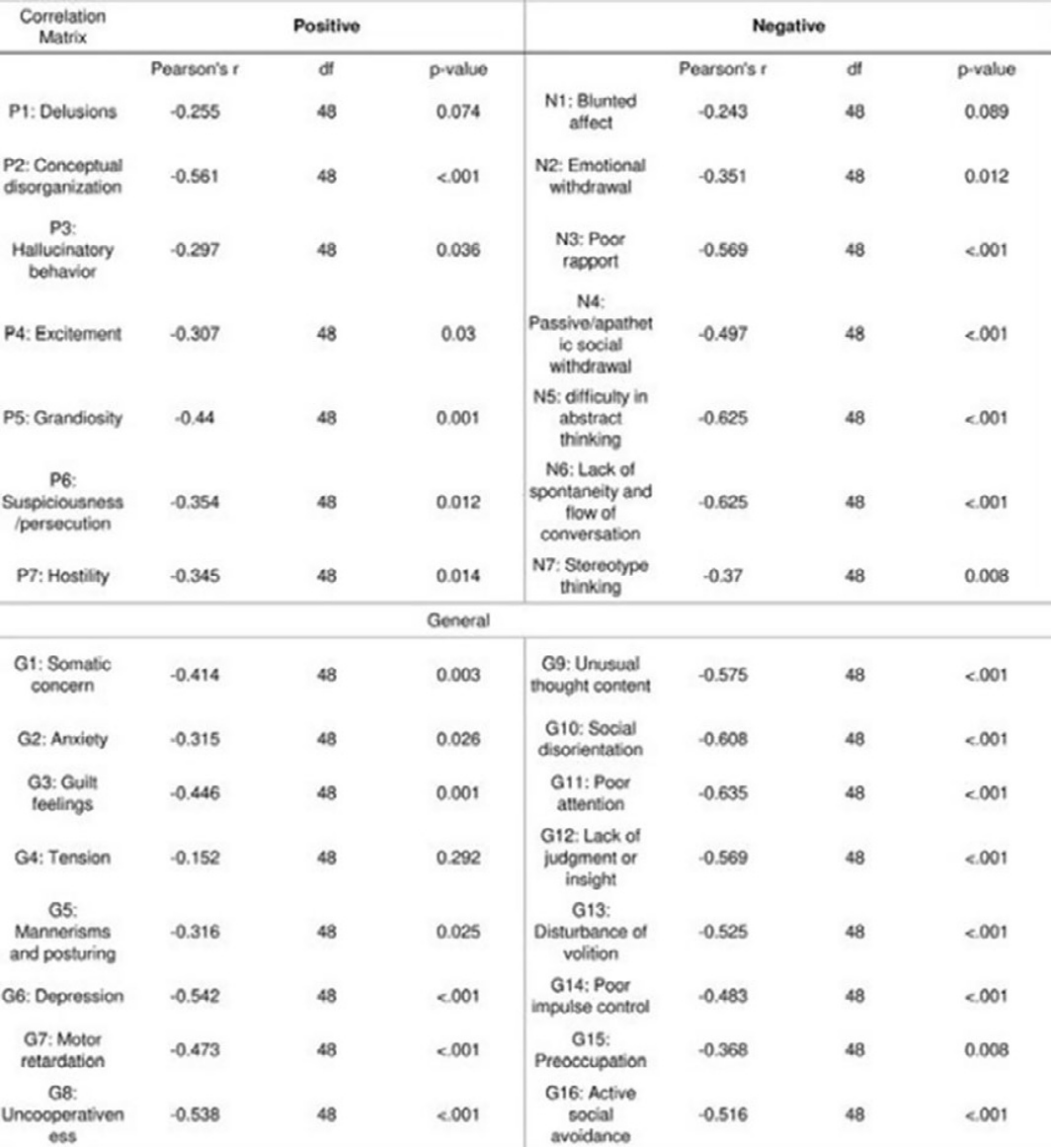

**Image 3:**

**Figure:**
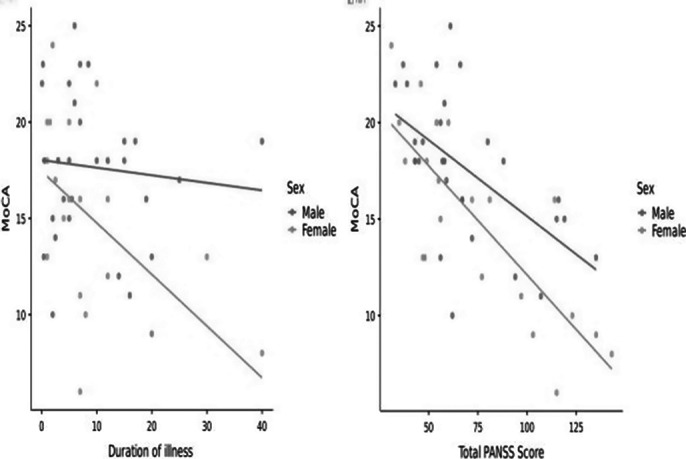

**Conclusions:**

We conclude that cognitive function significantly declines( executive function was the most and memory was not significantly impacted) with respect to increasing disease severity and duration of illness in long term psychosis.Most positive symptoms, excluding delusions,and negative symptoms (apart from blunted affect) and general symptoms (except tension), were significantly linked to cognitive decline.

**Disclosure of Interest:**

None Declared

